# Computational Prediction of the Therapeutic Mechanisms From the Turmeric‐Pueraria‐Coix Seed Botanical Formula in Rheumatoid Arthritis

**DOI:** 10.1002/fsn3.72503

**Published:** 2026-10-21

**Authors:** Weiya Lan, Qianwei Lu, Xudong Rui, Ke Li, Chaoji Su, Qiang Su, Yanchang Huang, Run Shi, Wei Chen, Fang Tang

**Affiliations:** ^1^ Graduate School Guizhou University of Traditional Chinese Medicine Guiyang Guizhou Province China; ^2^ Department of Joint Orthopedics The Second Affiliated Hospital of Guizhou University of Traditional Chinese Medicine Guiyang Guizhou Province China; ^3^ School of Health Management Guiyang Healthcare Vocational University Guiyang Guizhou Province China; ^4^ Department of Joint Orthopedics Nanyang Hospital of Traditional Chinese Medicine Nanyang Henan Province China; ^5^ Department of Oncology The First Affiliated Hospital of Nanjing Medical University Nanjing Jiangsu Province China; ^6^ Department of Neurology The Affiliated Jinyang Hospital of Guizhou Medical University Guiyang Guizhou Province China; ^7^ Department of Rheumatology and Immunology The Second Affiliated Hospital of Guizhou University of Traditional Chinese Medicine Guiyang Guizhou Province China

**Keywords:** coumestrol, dietary polyphenols, ferroptosis, machine learning, molecular dynamics, rheumatoid arthritis

## Abstract

Rheumatoid arthritis (RA) is characterized by progressive synovial inflammation and immune‐metabolic reprogramming. The polyphenol‐rich Turmeric‐Pueraria‐Coix seed (TPC) formula shows therapeutic potential against RA, though its multi‐target mechanisms remain uncharacterized. To explore how TPC modulates ferroptosis and oxidative stress in RA, we integrated systems nutrition network analysis with multicohort transcriptomic datasets to identify candidate targets. Ensemble machine learning algorithms and independent cohort validation prioritized key biomarkers. Molecular dynamics (MD) simulations evaluated the dynamic behavior and interaction persistence of polyphenol‐target complexes, and single‐cell in silico overexpression and knockout analyses characterized the regulatory consequences of perturbing these targets within RA synovial networks. Analysis identified 85 bioactive phytochemicals in the TPC formula, forming a polyphenol‐dominant matrix mapping to 40 RA targets. Algorithmic and cross‐cohort prioritization isolated six core genes (ALOX5, CD38, CDK1, EZH2, MAPK8, and PTGS2) linked to RA synovial transcriptomic patterns, with varying predictive performance across external cohorts. SHAP analysis highlighted PTGS2, CD38, and MAPK8; however, MD simulations prioritized CD38 and MAPK8 as stable targets of coumestrol, whereas PTGS2 exhibited less dynamic stability. Single‐cell perturbation analysis revealed similar regulatory responses following MAPK8 and PTGS2 modulation, whereas CD38 knockout produced a distinct network response. These findings link TPC‐derived polyphenols to CD38/MAPK8 signaling and ferroptosis in RA, providing testable molecular hypotheses for experimental validation.

## Graphical abstract

Overview of the integrated computational framework used to investigate the Turmeric–Pueraria–Coix seed formula in rheumatoid arthritis. Phytochemical and disease‐associated targets were integrated with ferroptosis and oxidative‐stress genes, multicohort transcriptomics, machine learning, structural modeling, and single‐cell in silico perturbation to prioritize candidate phytochemical–target relationships.

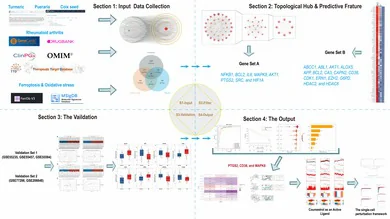

## 1 Introduction

Non‐communicable chronic diseases (NCDs) driven by chronic inflammation and metabolic dysfunction impose an escalating global health burden. Rheumatoid arthritis (RA) epitomizes these conditions, defined by chronic synovitis and progressive joint erosion (Noto et al. 2025). Recent systems biology insights suggest that RA pathogenesis involves not only immune dysregulation but also profound metabolic reprogramming within the synovial microenvironment (Jiang et al. 2025). Under the continuous stimulation of hypoxia and pro‐inflammatory factors, the energy metabolism patterns of fibroblast‐like synoviocytes (RA‐FLS) and macrophages undergo rearrangement, accompanied by the accumulation of local reactive oxygen species (ROS) and iron metabolism disorders, which subsequently trigger lipid peroxidation and pathological damage associated with ferroptosis (Aghakhani et al. 2022; Liu et al. 2024). Given that RA‐FLS, which exhibit tumor‐like proliferative characteristics, possess strong tolerance to ferroptosis (Wu et al. 2022), targeting ferroptosis‐related pathways in RA‐FLS has emerged as a potential strategy for limiting synovial hyperplasia (Bieri et al. 2024). In this context, bioinformatics analyses have identified ferroptosis‐related transcriptional signatures in RA and proposed several genes, including MAPK8, as candidate biomarkers or therapeutic targets (Li et al. 2023). More recently, integrated bioinformatics and single‐cell RNA‐sequencing analyses have characterized cuproptosis‐related genes across RA cell populations and examined their associations with immune‐related features and potential therapeutic targets (Li et al. 2025). Together, these studies illustrate the growing use of transcriptomic and computational approaches to investigate regulated cell‐death pathways in RA. Ultimately, translating these in silico‐derived RCD targets into clinical applications remains a key objective for advancing RA management.

In response to the escalating global burden of NCDs, targeted dietary modifications have emerged as a pivotal preventative and adjunctive strategy. Dietary polyphenols are plant‐derived bioactive compounds with reported anti‐inflammatory, antioxidant, and immunomodulatory activities (Yahfoufi et al. 2018; Zekrumah et al. 2023). Accumulating evidence indicates that polyphenols can modulate the inflammatory pathways and oxidative stress that characterize prevalent arthropathies, positioning them as promising nutritional agents for RA control (Sung et al. 2019; Oliviero et al. 2018). As a classic “medicine and food homology” botanical combination, the Turmeric‐Pueraria‐Coix seed (TPC) formula contains multiple classes of phytochemicals, including polyphenols (particularly flavonoids) and selected functional lipids. Modern research has established that its primary polyphenolic components, such as curcumin from turmeric and coumestrol from Pueraria, possess potent biological activities, including antioxidation, metabolic regulation, and the improvement of inflammatory polarization. These properties demonstrate tremendous potential in the daily dietary intervention and nutritional management of chronic inflammatory diseases like RA (Xu et al. 2022; Kou et al. 2025; Ouyang et al. 2025; Peng et al. 2021).

Despite these promising attributes, the specific multi‐target synergistic mechanisms by which the complex dietary mixture of TPC might modulate the immune‐metabolic network of RA remain to be systematically elucidated. Deciphering the pharmacodynamic basis of such complex, polyphenol‐rich functional foods presents a profound computational challenge. While traditional network pharmacology has been widely applied to decode the mechanisms of multi‐component natural products, its heavy reliance on static public databases fails to capture the spatiotemporal dynamic changes inherent to disease progression (Cui et al. 2025). Furthermore, conventional rigid molecular docking—often employed as a routine validation tool—inadequately accounts for the conformational flexibility of macromolecular targets and complex aqueous solvation effects. This methodological bottleneck frequently generates high false‐positive rates due to inaccurate evaluations of true binding free energies, thereby limiting its translational value in precision nutrition (Ibrahim et al. 2024; Che and Zhang 2025).

We integrated multicohort synovial transcriptomic analysis, interpretable machine learning, molecular docking, explicit‐solvent all‐atom molecular dynamics (MD) simulations, and single‐cell in silico perturbation to investigate molecular relationships between TPC‐derived phytochemicals and RA. Transcriptomic and machine‐learning analyses prioritized RA‐associated molecular features, with SHAP attribution quantifying their contributions to model classification (Wu et al. 2026; Brooks et al. 2025; Ullah et al. 2025). Molecular docking and MD simulations evaluated candidate phytochemical‐target interactions structurally and dynamically, whereas virtual overexpression and knockout analyses characterized the regulatory consequences of perturbing prioritized targets within RA synovial single‐cell networks. This integrated framework prioritizes phytochemical–target relationships and characterizes their transcriptomic, structural, and regulatory‐network features, providing testable molecular hypotheses for experimental validation.

## 2 Materials and Methods

### 2.1 Screening of Bioactive Phytochemicals and Target Prediction

To characterize the bioactive constituents, particularly dietary polyphenols, of the Turmeric‐Pueraria‐Coix seed (TPC) formula, phytochemical profiles were extracted from TCMSP and SymMap. Initial filtering prioritized compounds with oral bioavailability (OB) ≥ 30% and drug‐likeness (DL) ≥ 0.18. Molecular structures retrieved from PubChem were further filtered using a molecular‐weight threshold of < 500 Da (Lipinski et al. 2001). A secondary SwissADME screening retained molecules satisfying at least two criteria across five drug‐likeness models (Lipinski, Ghose, Veber, Egan, and Muegge). Finally, potential human targets were predicted via SwissTargetPrediction, retaining only those surpassing the median probability threshold. De‐duplicated targets were aggregated to construct the TPC “bioactive phytochemical‐potential target” network.

### 2.2 Acquisition of Disease‐Related Targets for Rheumatoid Arthritis (RA)

Pathogenic targets for rheumatoid arthritis (RA) were retrieved from GeneCards using a relevance score threshold of > 1.0. Additionally, all uniquely annotated RA targets were sourced from DrugBank, PharmGKB, OMIM, and TTD to ensure a comprehensive target set. Following UniProt‐standardized gene symbol mapping and redundancy removal, a consolidated RA target set was established. Finally, intersecting these with TPC‐derived bioactive phytochemical targets via Venn analysis yielded common regulatory nodes.

### 2.3 Construction and Mapping of Ferroptosis and Oxidative Stress‐Specific Gene Sets

To clarify TPC's impact on RA‐related ferroptosis and oxidative stress, human ferroptosis genes (FerrDb V2) and oxidative stress response sets (MSigDB: GO:0006979, WP) were integrated. Mapping these unique genes against “phytochemical‐disease” shared targets isolated a specific cohort for downstream topological analysis and dimensionality reduction.

### 2.4 Screening of Core Targets Based on Protein–Protein Interaction (PPI) Network

Mapping the intersecting targets to STRING 12.0 yielded a protein–protein interaction (PPI) network (medium confidence, > 0.4). After filtering isolated nodes, we visualized the assembly in Cytoscape (v3.10.4). Comprehensive topological screening was then performed via CytoNCA, utilizing the intersection of all available centrality metrics to isolate the core hubs, which were designated as Gene Set A.

### 2.5 Acquisition and Preprocessing of Gene Expression Data

Human synovial gene‐expression datasets were retrieved from NCBI GEO using the keywords “Rheumatoid Arthritis” AND (“Synovium” OR “Synovial tissue”). Datasets were selected to support RA–HC comparisons at the integrated cohort level, resulting in six included datasets (Table 1). The largest dataset, GSE89408 (152 RA and 28 HC samples), was used as the training cohort. The remaining datasets were integrated into two external validation cohorts according to platform compatibility: Validation Set 1 combined GSE55235, GSE55457, and GSE55584 (20 HC and 33 RA samples; *n* = 53), whereas Validation Set 2 combined GSE77298 and GSE206848 (14 HC and 18 RA samples; *n* = 32). Although GSE55584 contained RA samples only, it was not evaluated independently; instead, it was incorporated into the merged GPL96‐based Validation Set 1 before batch‐effect correction and subsequent cohort‐level validation. Following dataset‐specific preprocessing, expression matrices within each validation set were harmonized by common gene identifiers, merged, and subjected to batch‐effect correction before downstream validation analyses were performed on the integrated cohort. R‐based preprocessing (v4.5.1) employed GEOquery (v2.72.0) for data retrieval and edgeR (v4.0.16) for annotation. Noise reduction involved filtering sparsely expressed genes (CPM ≤ 1 in < 10 samples). Finally, we applied TMM normalization and log2 transformation (prior. count = 2) to generate the standardized LogCPM matrix.

**TABLE 1:** Baseline characteristics of the included GEO datasets.

DataSets	Platforms	Sample size		Organism	Tissue	Attribute
		Normal	RA			
GSE89408	GPL11154	28	152	*Homo sapiens*	Synovial membrane	Train
GSE55235	GPL96	10	10	*H. sapiens*	Synovial membrane	Validation1
GSE55457	GPL96	10	13	*H. sapiens*	Synovial membrane	Validation1
GSE55584	GPL96	0	10	*H. sapiens*	Synovial membrane	Validation1
GSE77298	GPL570	7	16	*H. sapiens*	Synovial membrane	Validation2
GSE206848	GPL570	7	2	*H. sapiens*	Synovial membrane	Validation2

### 2.6 Machine Learning‐Based Feature Variable Screening

We implemented an ensemble framework in R leveraging 15 machine‐learning algorithms to prioritize RA‐associated transcriptomic features within synovial tissue. The pipeline integrated diverse methods, including regularized regressions (Lasso, Ridge, and Elastic Net), discriminant analyses (LDA and QDA), ensemble learners (RF, GBM, XGBoost, AdaBoost, and glmBoost), and standard classifiers (SVM, Stepwise GLM, plsRglm, Naive Bayes, and KNN). A two‐stage protocol was implemented for model development. First, gene expression dimensionality was reduced using established feature selection methods (e.g., LASSO and Random Forest), followed by the training of various classifiers on the retained features. This combinatorial approach yielded 175 distinct modeling strategies (summarized in Figure 1). To balance model interpretability with complexity, the final feature subsets were constrained to include between 3 and 15 variables. Model‐specific internal cross‐validation or resampling procedures were used for parameter tuning where applicable. To mitigate the marked class imbalance in the training cohort (152 RA vs. 28 HC), SMOTE was incorporated during training of the plsRglm model and was not applied to either external validation cohort. Precision–Recall curves and AUC‐PR values were additionally calculated to complement ROC‐based performance evaluation under class imbalance. These procedures were intended to mitigate, rather than eliminate, the uncertainty associated with the uneven class distribution. During final model evaluation, priority was given to algorithmic stability against the multicollinearity often present in high‐dimensional transcriptomic data, rather than relying exclusively on maximum predictive scores. Model performance was compared using the predefined evaluation framework, and the retained features from the selected plsRglm model were designated as Gene Set B.

**FIGURE 1:**
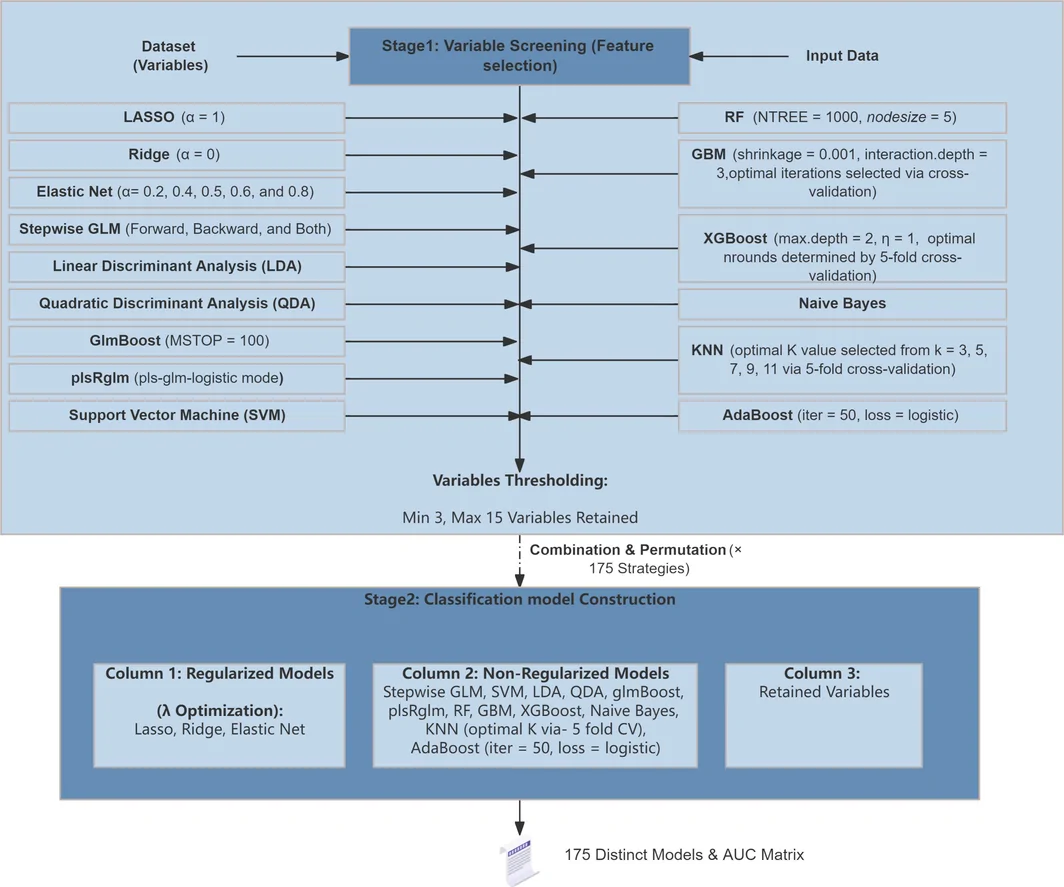
Workflow of the integrated machine‐learning classification modeling strategy.

### 2.7 Establishment of Core Targets and Construction of a Nomogram Model

To mitigate multicollinearity and enhance biological relevance, we intersected machine learning features (Gene Set B) with topological core nodes (Gene Set A). Across the two batch‐corrected external validation cohorts (total *n* = 85), Wilcoxon rank‐sum tests were used to assess the prioritized candidate features (*p* < 0.05), which were further refined against training set DEGs to identify core TPC‐associated candidate targets. To reduce multicollinearity among the six prioritized targets, LASSO regression was applied for further feature selection. A multivariate nomogram based on the retained features and training‐set coefficients was constructed as an exploratory visualization of their joint contribution to RA–HC classification. ROC, calibration, and decision‐curve analyses were used to characterize model performance within the analyzed transcriptomic datasets.

### 2.8 Model Interpretation and Feature Attribution Based on the SHAP Algorithm

The SHAP (SHapley Additive exPlanations) framework was used to quantify the contribution of individual features to model predictions. Feature importance was ranked according to mean absolute SHAP values. Global feature contributions were visualized using beeswarm and bar plots, while dependence plots were used to examine nonlinear relationships between gene expression and model output. Individual‐level waterfall plots were generated to visualize sample‐specific feature contributions to RA–HC classification.

### 2.9 Secondary Screening of Bioactive Phytochemicals and Molecular Docking Validation

Receptors accounting for > 80% cumulative SHAP contribution were prioritized for docking. TPC components were further screened according to predefined predicted ADMET and drug‐likeness criteria: high GI absorption, non‐inhibition of CYP3A4, ≤ 1 Lipinski violation, bioavailability score ≥ 0.55, and zero PAINS alerts. Ligand structures (PubChem) and receptor crystals (RCSB PDB) were processed using AutoDockTools (v1.5.7) to remove redundant chains and assign Gasteiger charges. For blind docking via QuickVina‐W (gluedocking R package), the grid box was centered on the geometric center of the target protein, encompassing the entire protein topology with a 5 Å buffer, and executed with an exhaustiveness of 8. Top‐ranked complexes, identified by binding affinity and bioactive pocket alignment, proceeded to molecular dynamics (MD) simulation.

### 2.10 Molecular Dynamics Simulation and Binding Free Energy Calculation

Using GROMACS 2025.2, we performed 100 ns all‐atom MD simulations on docked complexes. Proteins and ligands were parameterized with AMBER99SB‐ILDN and GAFF, respectively. After neutralizing the system in a TIP3P‐solvated cubic box, we conducted two‐stage energy minimization (steepest descent and conjugate gradient) until maximal force reached < 1000 kJ/mol/nm. Sequential 2‐ns NVT and NPT equilibrations (2‐fs timestep) preceded the 100‐ns NPT production run. Trajectory analyses were performed to calculate the RMSD, RMSF, Rg, SASA, and hydrogen bonding profiles. System convergence was evaluated by monitoring the stabilization of the Cα root‐mean‐square deviation (RMSD). Equilibration was considered achieved when conformational fluctuations remained within a narrow range (e.g., amplitude variations < 0.2 nm) following the initial structural adjustment. MM/PBSA calculations were restricted to frames sampled from the relatively equilibrated portion of the trajectories. Structural snapshots were extracted at regular intervals from the final 30 ns of the production trajectories to characterize representative conformational states sampled during the relatively equilibrated portion of each trajectory. Representative binding poses were visualized in 2D and 3D using Maestro and PyMOL, respectively; differences in hydrogen‐bond annotation between the two visualization platforms were documented where applicable. Given that each complex was evaluated using a single 100‐ns production trajectory, the MD analyses were interpreted as exploratory assessments of trajectory‐dependent conformational behavior rather than definitive measurements of long‐term thermodynamic or kinetic stability.

### 2.11 Single‐Cell Transcriptomic In Silico Perturbation Analysis

To examine the regulatory consequences of candidate targets in RA synovial cells, single‐cell RNA‐sequencing data (GSE200815) were processed using Seurat and integrated via Harmony. Raw count matrices from annotated RA cells underwent in silico perturbation. Virtual overexpression was simulated using scTenifold (Osorio et al. 2020). Following quality control and counts per million (CPM) normalization, 1000 genes were retained based on expression dispersion. Ten gene‐regulatory networks were constructed by sampling 500 cells per network, followed by tensor decomposition to generate a denoised baseline network. Target overexpression was simulated via twofold amplification of outgoing regulatory edges, preserving directionality. Virtual knockouts were independently simulated using scTenifoldKnk with identical network size and dimensionality settings (Osorio et al. 2022). Baseline and perturbed networks were compared via manifold alignment, and gene‐level regulatory displacement was quantified from the low‐dimensional representations. *p* values were adjusted using the Benjamini–Hochberg method, with a false discovery rate (FDR) < 0.05 defining statistical significance.

## 3 Results

### 3.1 Screening of Bioactive Phytochemicals and Construction of the Target Network for the TPC Formula

Applying screening criteria for oral bioavailability (OB ≥ 30%), drug‐likeness (DL ≥ 0.18), and molecular weight (MW < 500 Da), we identified 85 bioactive phytochemicals—predominantly comprising dietary polyphenols, flavonoids, and allied functional lipids—and 687 predicted downstream targets from the TPC formula. Integration of five curated disease databases (GeneCards, DrugBank, PharmGKB, OMIM, and TTD) yielded 4275 rheumatoid arthritis (RA)–related targets. Intersection analysis mapped 385 overlapping targets between the formula and the disease (Figure 2A). Topological analysis identified 2‐monoolein (Coix seed), coumestrol (Pueraria), and bisdemethoxycurcumin (Turmeric) as three of the highest degree phytochemical nodes.

**FIGURE 2:**
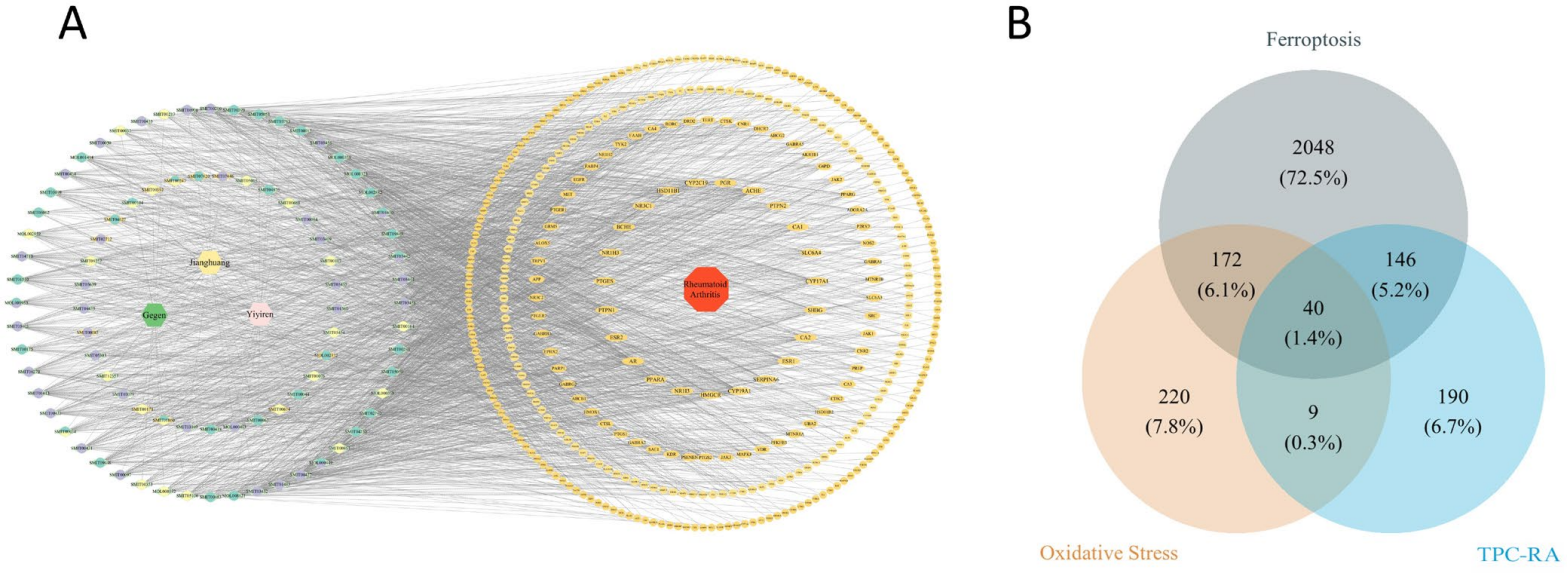
Network synthesis and gene identification for the TPC formula. (A) Integrated network mapping TPC phytochemicals against RA pathology targets. (B) Venn diagram delineating the convergence of ferroptosis and oxidative stress within TPC‐RA shared pathways.

### 3.2 Identification of Ferroptosis and Oxidative Stress‐Specific Targets

Mapping the shared phytochemical–RA targets to the ferroptosis and oxidative‐stress gene sets identified 40 intersecting candidate targets (Figure 2B).

### 3.3 Screening of Core Biomarkers Based on Network Topology and Machine Learning

To predict primary targets for the TPC formula, we integrated network topology with machine learning. A STRING‐derived protein–protein interaction (PPI) network comprising the 40 pre‐identified targets was analyzed via CytoNCA, revealing eight hub nodes (Gene Set A): *NFKB1, BCL2, IL6, MAPK8, AKT1, PTGS2, SRC*, and *HIF1A* (Figure 3A; Figure S1). Concurrently, 175 algorithmic combinations were evaluated for feature selection within the GEO training set (GSE89408, Figure S2A,B). The stability of model performance and the effectiveness of batch correction were subsequently assessed across two consolidated validation sets (Figure 3B–E). Among the tested approaches, a partial least squares‐generalized linear model (plsRglm) with 10‐fold cross‐validation yielded the highest predictive performance (AUC = 0.837). PR analysis yielded AUC‐PR values of 0.998, 0.930, and 0.690 in the training set, Validation Set 1, and Validation Set 2, respectively (Figure S3). AUC‐PR was lower in Validation Set 2 than in the training set and Validation Set 1. Based on this optimization, the model identified 15 core feature genes (Gene Set B), including *ABCC1, ABL1, AKT1, ALOX5, APP, BCL2, CA3, CAPN2, CD38, CDK1, ERN1, EZH2, G6PD, HDAC2*, and *HDAC6* (Figure 3F).

**FIGURE 3:**
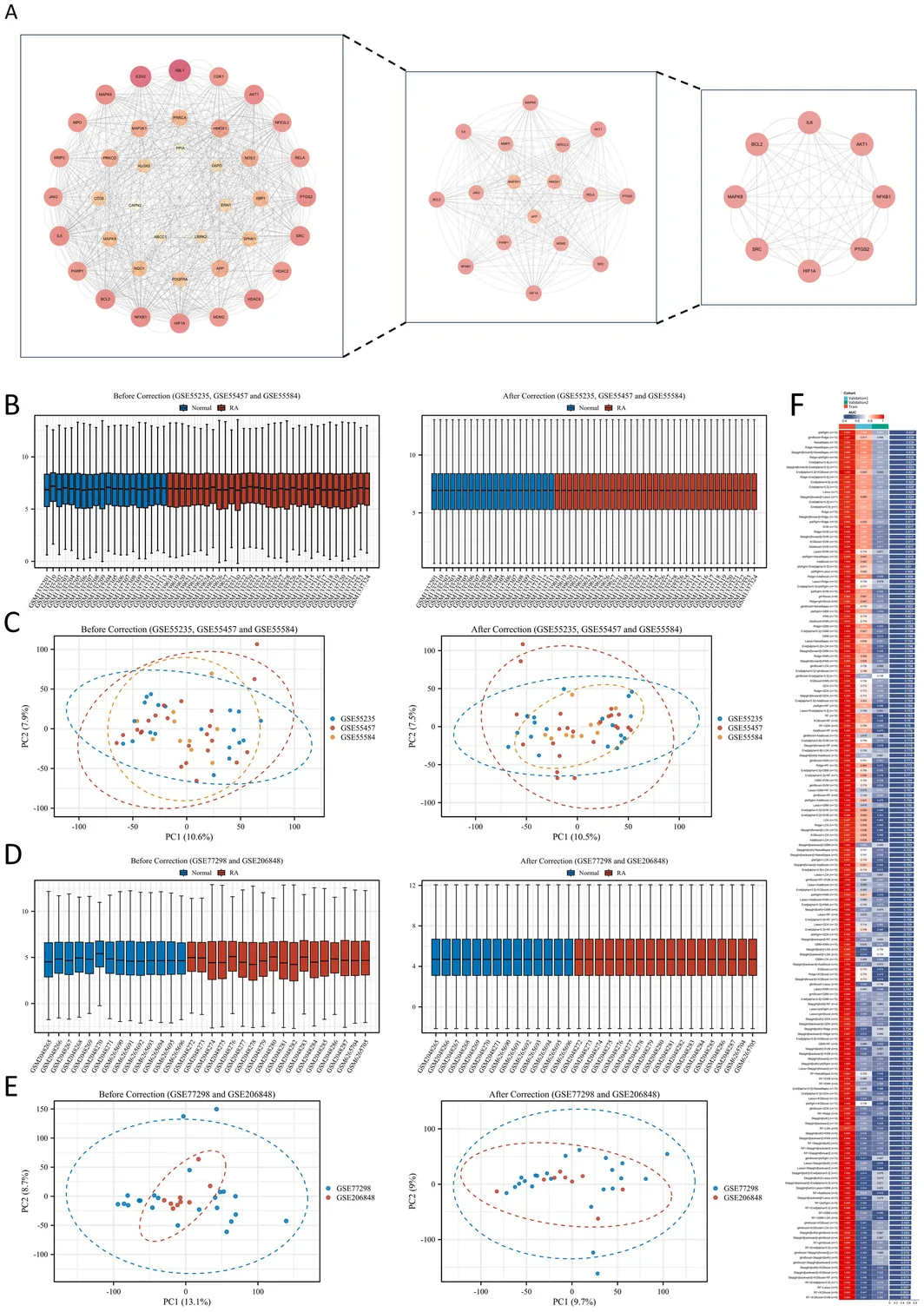
Multidimensional cross‐validation and machine learning‐driven biomarker identification. (A) CytoNCA‐derived core topological nodes from the PPI network. (B–E) Batch effect correction and normalization assessment for Validation Sets 1 and 2, visualized via boxplots (B, D) and PCA (C, E). (F) Integrated feature screening and performance benchmarking across 175 machine learning ensemble combinations.

### 3.4 Cross‐Cohort External Evaluation of Core Candidate Genes

To assess the cross‐cohort reproducibility of the prioritized features within synovial tissue, differential expression analysis of the intersecting genes from Gene Set A and Gene Set B was performed in the merged external Validation Set 1 (GSE55235, GSE55457, and GSE55584) and Validation Set 2 (GSE77298 and GSE206848). Following batch effect correction using the ComBat algorithm, PCA was used to visualize sample distributions after ComBat batch‐effect correction (Figure S4A,B). Through the Wilcoxon rank‐sum test, we identified 6 core genes with statistically significant differential expression (*p* < 0.05) in the external evaluation cohorts: *ALOX5, CD38, CDK1, EZH2, MAPK8*, and *PTGS2* (Figure 4A,B).

**FIGURE 4:**
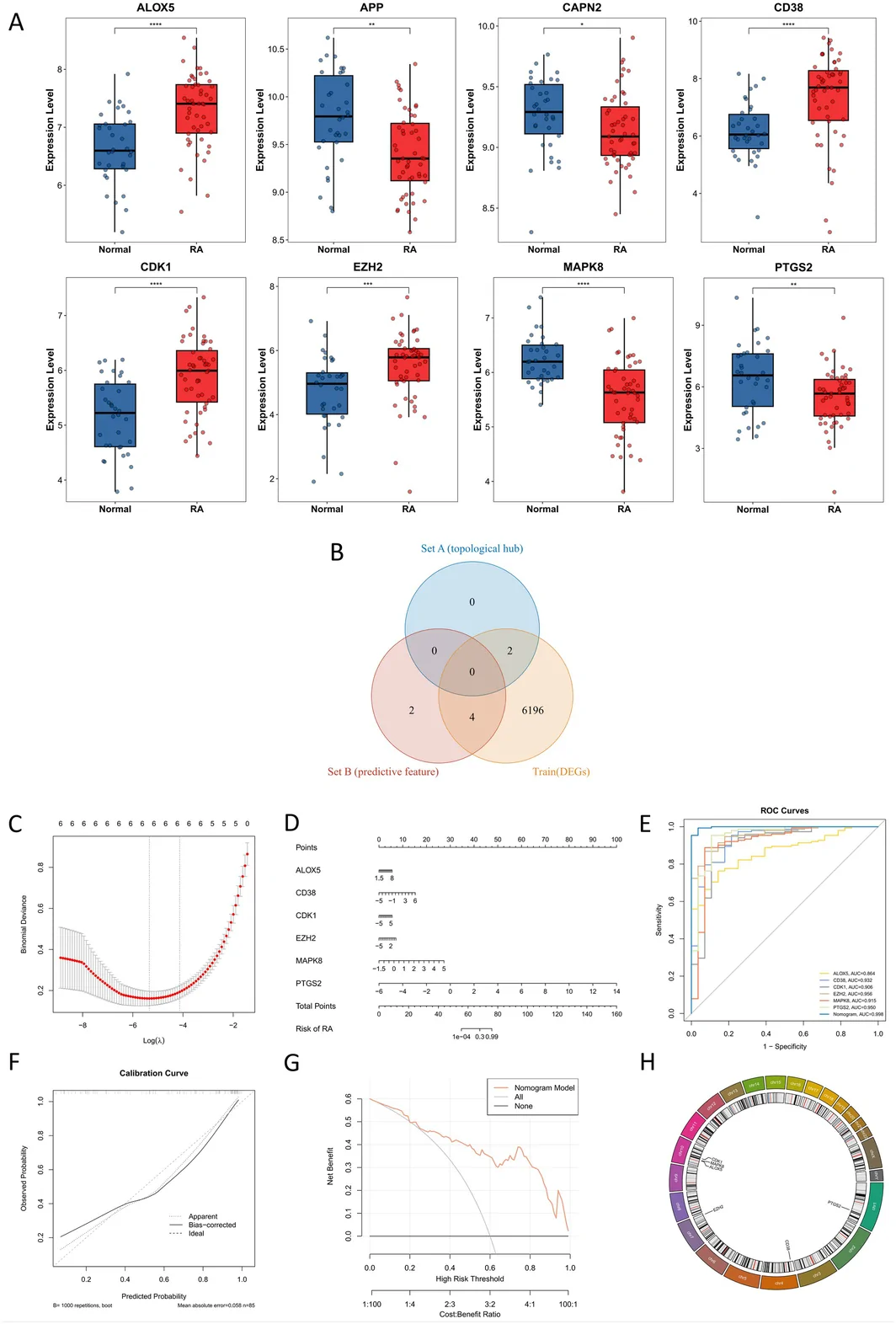
External evaluation and nomogram construction. (A) Differential core gene expression across validation cohorts (*p < 0.05 to ****p < 0.0001). (B) Integrated strategy for biomarker extraction. (C) LASSO‐based feature selection and cross‐validation. (D) Exploratory nomogram incorporating the retained core features. (E) ROC analysis evaluating model and biomarker performance. (F) Calibration curve assessing model fit. (G) Decision‐curve analysis of model‐based net benefit across threshold probabilities. (H) Circos plot illustrating chromosomal mapping and co‐expression patterns.

### 3.5 Construction and Performance Evaluation of the Nomogram Predictive Model

To reduce potential multicollinearity among the six prioritized targets, LASSO regression was applied for further feature selection, and an exploratory nomogram was constructed to visualize their joint contribution to RA–HC classification (Figure 4C,D). PTGS2 showed the largest model coefficient, followed by MAPK8. The nomogram achieved an AUC of 0.998 within the analyzed dataset, while the individual features each showed AUC values above 0.85 (Figure 4E). The calibration curve showed agreement between predicted classification probabilities and observed class labels within this dataset (Figure 4F). Decision‐curve analysis was additionally performed as an exploratory assessment of model‐based net benefit across threshold probabilities (Figure 4G). Finally, the chromosomal locations and coexpression relationships of the six core genes were visualized using a Circos plot (Figure 4H).

### 3.6 Interpretability and Feature Attribution of the Machine Learning Model Based on the SHAP Algorithm

SHAP analysis ranked PTGS2, CD38, and MAPK8 as the three features with the largest contributions to model output (Figure 5A). The high expression (yellow scatter points in the figure) of *PTGS2, CD38*, and *MAPK8* was primarily distributed in the positive SHAP value region, indicating that higher expression of these genes was associated with positive SHAP contributions toward classification into the RA group. Permutation feature‐importance analysis similarly ranked PTGS2, CD38, and MAPK8 among the most influential model features (Figure 5B). PTGS2, CD38, and MAPK8 collectively accounted for > 80% of the cumulative absolute SHAP contribution (Figure 5C), whereas ALOX5, EZH2, and CDK1 showed lower contributions.

**FIGURE 5:**
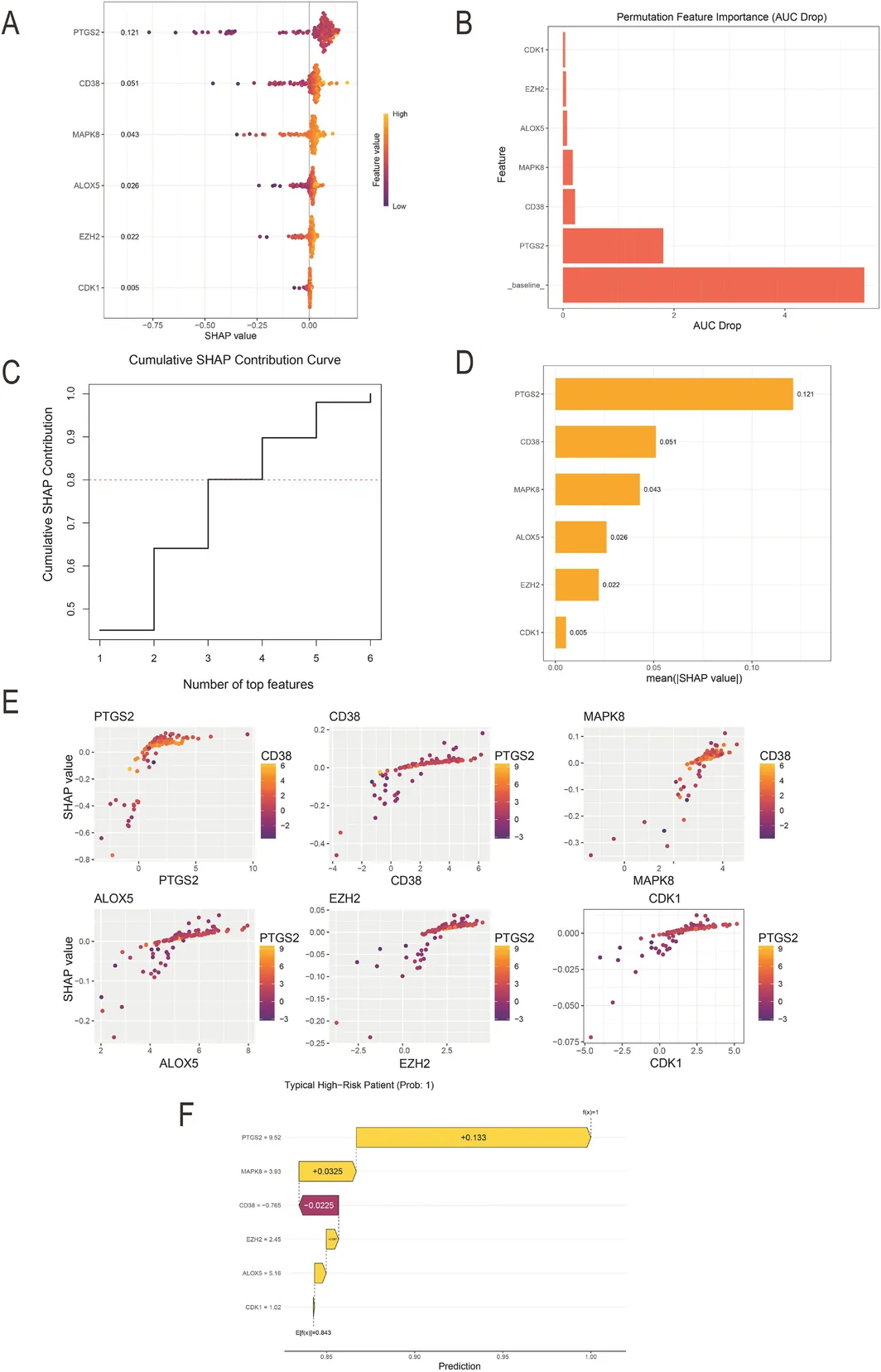
Model interpretability and feature attribution based on the SHAP analysis. (A) SHAP summary beeswarm plot of core biomarkers. (B) Feature importance analysis based on permutation. (C) Cumulative SHAP contribution curve of core genes. (D) Feature importance bar chart of mean absolute SHAP values. (E) SHAP dependence plots revealing the nonlinear relationship between gene expression levels and RA classification probability. (F) SHAP waterfall plots illustrating individual‐sample contributions to RA–HC classification.

In the mean absolute SHAP value (Mean |SHAP|) bar chart, the global importance of *PTGS2* ranked first (mean |SHAP| = 0.121), followed by *CD38* and *MAPK8* (Figure 5D). SHAP dependence plots showed nonlinear relationships between gene expression and model‐predicted RA classification probability (Figure 5E). In the illustrated sample with a high predicted probability of RA classification, elevated PTGS2 expression (expression value = 9.52) produced the largest positive SHAP contribution (+0.133). Together with MAPK8 and EZH2, these features increased the model‐predicted probability of classification into the RA group (Figure 5F).

### 3.7 Secondary ADMET Screening of Bioactive Components

PTGS2, CD38, and MAPK8 were selected for subsequent molecular‐docking analysis based on the SHAP feature attribution results. Candidate TPC phytochemicals targeting these proteins were further screened according to the predefined ADMET and drug‐likeness criteria. Ten compounds satisfied all specified criteria, including coumestrol (Table 2). Complete SwissADME outputs are provided in Data S1.

**TABLE 2:** ADMET and oral safety prediction results of the core candidate phytochemicals from the TPC formula.

Molecule	Canonical SMILES	GI absorption	CYP3A4 inhibitor	Lipinski violations	Bioavailability score	PAINS alerts
Coumestrol	Oc1ccc2c(c1)oc(=O)c1c2oc2c1ccc(c2)O	High	No	0	0.55	0
Turmeronol A	CC(=CC(=O)C[C@@H](c1ccc(c(c1)O)C)C)C	High	No	0	0.55	0
α‐Monoolein	CCCCCCCC/C=C\CCCCCCCC(=O)OC[C@@H](CO)O	High	No	0	0.55	0
Coixol	COc1ccc2c(c1)oc(=O)[nH]2	High	No	0	0.55	0
Anethole	C/C=C/c1ccc(cc1)OC	High	No	0	0.55	0
Linalool	C=CC(CCC=C(C)C)(O)C	High	No	0	0.55	0
Turmeronol B	CC(=CC(=O)CC(c1ccc(cc1O)C)C)C	High	No	0	0.55	0
Zedoarondiol	CC(=C1C[C@H]2[C@H]([C@@](CC1 = O)(C)O)CC[C@@]2(C)O)C	High	No	0	0.55	0
Nonanoic Acid	CCCCCCCCC(=O)O	High	No	0	0.85	0
2‐Monoolein	CCCCCCCC/C=C\CCCCCCCC(=O)OC(CO)CO	High	No	0	0.55	0

### 3.8 Molecular Docking and All‐Atom Molecular Dynamics (MD) Simulation Evaluation

To further examine the prioritized docking poses under an explicit‐solvent environment, coumestrol was subjected to 100‐ns MD simulations with CD38, MAPK8, and PTGS2. Within the sampled trajectories, coumestrol retained recurrent contacts with CD38 residues ASP217 and LYS214 and with MAPK8 residues ASN156 and ASP151 (Figure 6B–D). RMSD, RMSF, SASA, Rg and hydrogen‐bond analyses were used to characterize trajectory‐dependent structural fluctuations and interaction persistence (Figure 6E–I). MM/PBSA calculations yielded binding‐energy estimates of −113.35 and −129.83 kJ/mol for the CD38 and MAPK8 complexes, respectively (Figure 6J), while the free‐energy landscapes illustrated the conformational regions sampled during the trajectories (Figure 6K). In contrast, the PTGS2 complex showed loss of the initial docking pose within the 100‐ns simulation window. These observations describe the relative dynamic behavior of the three complexes within the sampled trajectories and should not be interpreted as evidence of long‐term thermodynamic stability or as excluding later dissociation of the CD38 or MAPK8 complexes.

**FIGURE 6:**
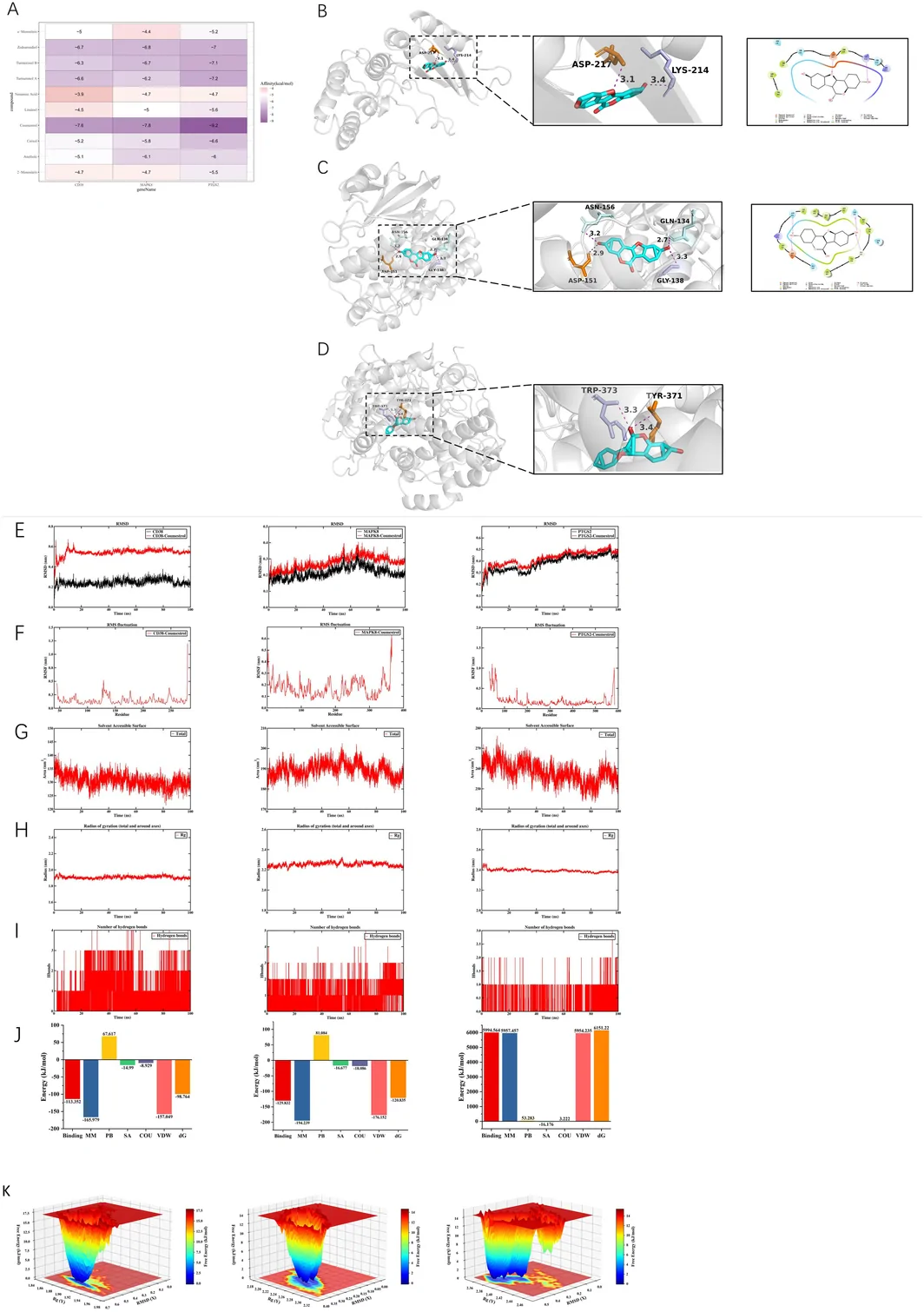
Molecular docking and 100 ns all‐atom MD simulation profiling. (A) Heatmap of predicted molecular‐docking affinities for phytochemical–protein complexes. (B–D) 3D and 2D interaction diagrams of representative coumestrol conformations sampled with CD38 (B), MAPK8 (C), and PTGS2 (D). Note that the 2D interaction map for the PTGS2 complex is absent due to a lack of detectable canonical hydrogen‐bonding networks in its initial static pose. (E–I) Trajectory evolution of structural metrics: (E) RMSD, (F) RMSF, (G) SASA, (H) Rg, and (I) hydrogen bond frequency. (J) MM/PBSA‐derived binding‐energy decomposition and (K) Gibbs free energy landscapes (FEL).

### 3.9 Single‐Cell In Silico Perturbation of Prioritized Targets

Virtual overexpression and knockout analyses assessed the regulatory consequences of prioritized targets in RA synovial single‐cell transcriptomic data (Figure 7). Quality control and feature selection retained 30,057 cells and 1000 genes for overexpression analysis. MAPK8 and PTGS2 comprised 912 and 908 perturbable outgoing edges, respectively, whereas CD38 was absent from the corresponding overexpression network. Virtual overexpression of MAPK8 induced significant regulatory displacement of MT‐ATP6 and MT‐ND4 (FDR = 9.66 × 10^−9^ and 3.06 × 10^−8^, respectively). PTGS2 overexpression produced a similar pattern, retaining significant displacement of MT‐ATP6 and MT‐ND4 (FDR = 2.32 × 10^−11^ and 1.00 × 10^−8^, respectively; Figure 7A,B; Figure S5 and Table S1).

**FIGURE 7:**
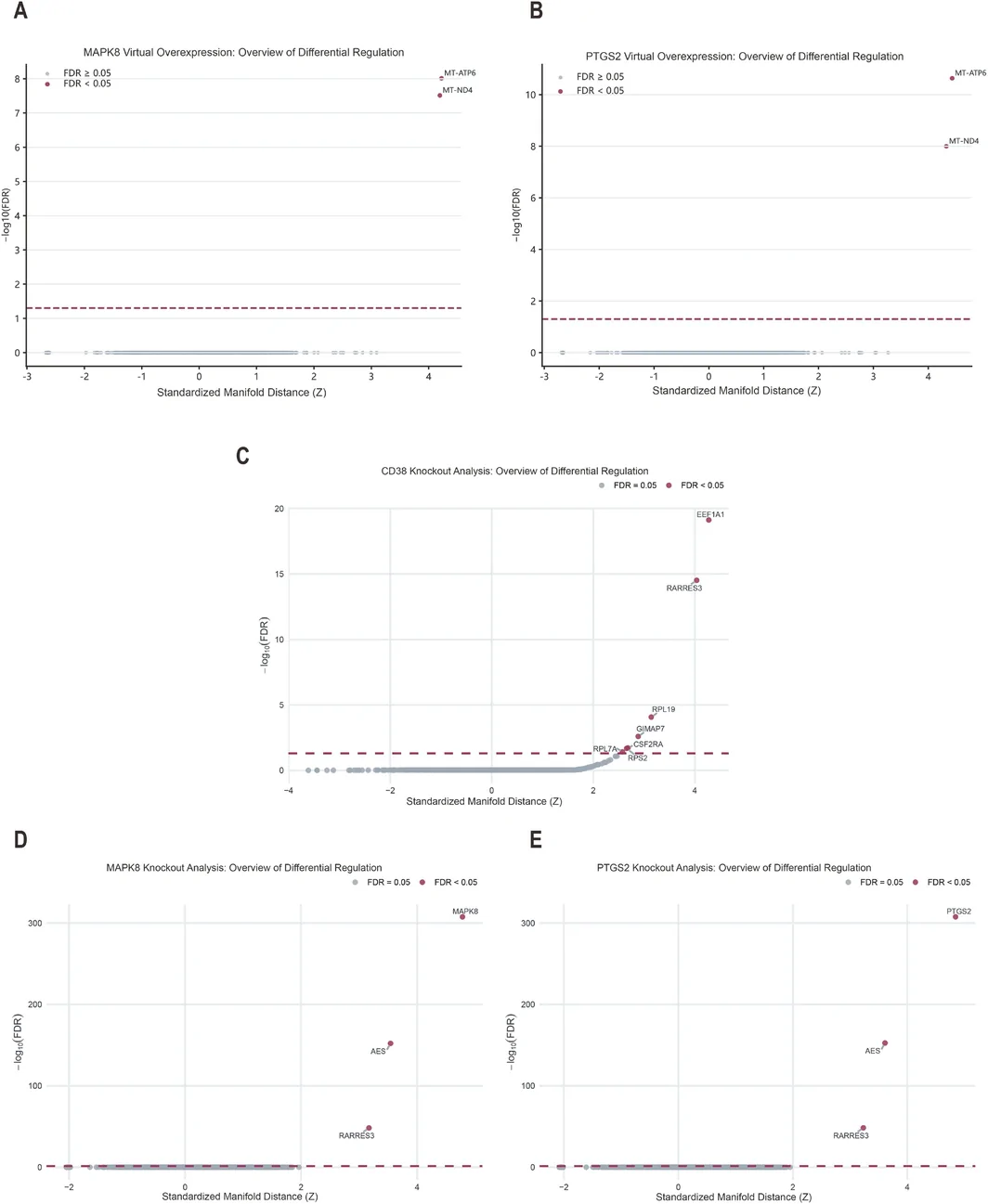
In silico gain‐ and loss‐of‐function perturbation of prioritized targets in RA synovial cells. (A) MAPK8 overexpression (OE) differential‐regulation scatter plot. (B) PTGS2 OE differential‐regulation scatter plot. (C) CD38 knockout (KO) differential‐regulation scatter plot. (D) MAPK8 KO differential‐regulation scatter plot. (E) PTGS2 KO differential‐regulation scatter plot.

In virtual knockout analyses, CD38 deletion triggered seven significant regulatory responses: EEF1A1, RARRES3, RPL19, GIMAP7, CSF2RA, RPS2, and RPL7A (FDR < 0.05). Excluding the perturbed targets, MAPK8 and PTGS2 knockouts both displaced AES and RARRES3 (Figure 7C–E; Figure S6 and Table S1). These analyses demonstrate distinct network responses following CD38 loss, alongside partially convergent responses to MAPK8 and PTGS2 modulation.

## 4 Discussion

The pathogenesis of RA involves complex immune–metabolic reprogramming and intersects with inflammatory and oxidative‐stress pathways common to other major NCDs. Persistent hypoxia and inflammation reshape synovial metabolism and contribute to fibroblast‐like synoviocyte proliferation and joint destruction (Henry and O'Neill 2025; Xie et al. 2026). These features have encouraged interest in dietary and botanical interventions containing bioactive polyphenols as potential adjunctive strategies (Zhou et al. 2022). Using an integrated systems nutrition and computational biology framework, this study identified 85 candidate bioactive phytochemicals in the TPC formula. Among these constituents, coumestrol and curcuminoid‐related polyphenols emerged as prominent candidates in the subsequent mechanistic analyses, while functional lipids such as 2‐monoolein represented additional components with potentially distinct biological roles.

Polyphenols regulate inflammatory and oxidative‐stress pathways in a context‐dependent manner. Curcumin suppresses NF‐κB‐associated inflammatory signaling while enhancing Nrf2‐dependent antioxidant responses across various inflammatory settings (Kou et al. 2023). Conversely, curcumin‐based photodynamic therapy promotes ferroptosis in RA fibroblast‐like synoviocytes by suppressing the Nrf2/xCT/GPX4 axis (Sun et al. 2025). Coumestrol, a Pueraria‐derived dietary phytoestrogen, drives estrogen receptor β‐mediated metabolic effects (Park et al. 2023). In IL‐1β‐stimulated primary rat chondrocytes, coumestrol attenuates matrix‐degrading enzymes, iNOS, COX‐2/PGE2, and inflammatory cytokine responses (You et al. 2017). Similarly, in LPS‐stimulated RAW264.7 macrophages, coumestrol and associated soy‐leaf coumestans reduce NO, PGE2, and ROS production (Li et al. 2017). These experimental profiles substantiate our computational prioritization of coumestrol as a modulator of oxidative stress and ferroptosis‐related networks in RA.

Functional lipids may contribute through pathways distinct from the predicted protein interactions prioritized for coumestrol. In the present docking analysis, 2‐monoolein showed weaker predicted affinities for the selected targets. Previous studies have implicated Coix seed‐derived lipids in modulation of the gut microbiota, butyrate production, and the gut–joint axis in inflammatory arthritis (Ouyang et al. 2025; Bai et al. 2025), while broader evidence suggests that dietary lipids can influence microbial and short‐chain‐fatty‐acid metabolism (Ma et al. 2025; Zaiss et al. 2021; Yu et al. 2022; Yoneda et al. 2025). Because microbiome and metabolomic data were not examined in this study, these mechanisms should be regarded as contextual hypotheses rather than direct findings.

Prioritized phytochemical‐target interactions converge on ferroptosis and oxidative‐stress pathways within RA synovial lesions. In the RA microenvironment, iron accumulation and reactive oxygen species (ROS) drive lipid peroxidation via Fenton chemistry, precipitating ferroptotic damage (Zhao, Dong, et al. 2024). Dietary polyphenols modulate these processes by regulating oxidative stress and iron homeostasis (Lesjak et al. 2022; Živanović et al. 2024). Crucially, ferroptosis drives cell‐specific consequences in RA: ferroptotic injury in macrophages or chondrocytes amplifies inflammation, whereas inducing ferroptosis in proliferative fibroblast‐like synoviocytes (RA‐FLS) restrains synovial hyperplasia (Cui et al. 2025; Lee et al. 2025; He et al. 2023). By mapping the regulatory consequences of upstream inflammatory and metabolic targets across RA synovial single‐cell networks, our computational framework provides a molecular basis for exploring these cell‐dependent ferroptotic responses.

Machine learning and SHAP attribution prioritized PTGS2, CD38, and MAPK8 among RA‐associated features, with structural analyses further identifying CD38 and MAPK8 as stable coumestrol targets. As a major NAD^+^‐consuming ectoenzyme, CD38 depletes the cellular NAD^+^ pool during inflammation, suppressing NAD^+^‐dependent sirtuin activity and altering metabolic‐inflammatory signaling (Zeidler et al. 2022). This CD38‐mediated NAD^+^ depletion increases ferroptosis susceptibility (Ma et al. 2022) by disrupting NADPH and glutathione homeostasis, thereby impairing cellular antioxidant capacity and GPX4‐dependent lipid‐peroxide control (Ma et al. 2022; Chen et al. 2025; Zhao et al. 2026). Our MD simulations demonstrated persistent coumestrol association with the CD38 binding region, providing a structural rationale for coumestrol‐mediated modulation of CD38 activity and downstream ferroptotic networks (Peclat et al. 2020).

MAP K8 is a stress‐signaling hub involved in oxidative‐stress responses. Its aberrant activation has been associated with MMP regulation and joint damage in RA (Kanai et al. 2020). The observed retention of coumestrol within the predicted MAPK8 binding region during the sampled trajectory supports prioritization of MAPK8 as a candidate interaction partner. Nevertheless, the present analysis does not demonstrate direct inhibition of MAPK8 activity or establish that coumestrol alters MAPK8‐dependent signaling in RA cells.

Classical ferroptosis‐related proteins such as GPX4 and SLC7A11 were not retained in the final feature set. This may partly reflect the penalization of correlated variables during dimensionality reduction. The resulting pattern may also indicate that the transcriptomic signals captured by the present model are concentrated in upstream inflammatory and metabolic pathways rather than in terminal ferroptosis effectors. CD38 and MAPK8 may therefore represent upstream candidates that warrant further testing for their potential influence on ferroptosis susceptibility. Such effects, if present, could occur indirectly through changes in the inflammatory and metabolic environment rather than through direct regulation of terminal ferroptosis machinery.

Although PTGS2 (COX‐2) contributed the highest predictive weight in the SHAP analysis and has been reported as a ferroptosis‐associated marker (He et al. 2023), the PTGS2–coumestrol complex lost its initial docking pose during the sampled 100‐ns trajectory. This discrepancy highlights the limitation of relying on static docking scores alone when receptor flexibility and explicit‐solvent effects are considered (Furse et al. 2006; Pantsar and Poso 2018; Rouzer and Marnett 2020; Eberhardt and Forli 2023). Within the sampled trajectory, the predicted PTGS2 interaction was less persistent than those observed for CD38 and MAPK8. PTGS2 was therefore assigned a lower priority as a direct coumestrol interaction candidate in the present computational analysis. Because this comparison was based on a single trajectory for each complex, it should be interpreted as a relative prioritization rather than as the exclusion of PTGS2 as a potential interaction target.

Single‐cell in silico perturbation delineated target‐specific network responses. Virtual overexpression of MAPK8 and PTGS2 converged on regulatory displacement of MT‐ATP6 and MT‐ND4, whereas knockout of both targets displaced AES and RARRES3. Conversely, CD38 knockout produced a broader and distinct regulatory pattern. The convergence between MAPK8 and PTGS2 indicates that distinct upstream perturbations can propagate toward shared downstream regulatory states. Furthermore, the recurrent displacement of mitochondrial‐encoded genes following overexpression implicates these targets in modulating mitochondrial network organization. Integrating multicohort transcriptomics, ensemble machine learning, structural simulations, and single‐cell in silico perturbations establishes a multidimensional framework for prioritizing phytochemical‐target interactions (Brooks et al. 2025; Liu et al. 2017; Hong et al. 2025). This approach bridges structural dynamics with network‐level regulatory consequences, providing a robust foundation for mechanistic discovery in RA (Zhao, Che, et al. 2024).

However, this study has several limitations inherent to its computational nature. First, in silico ADMET predictions cannot fully account for the absorption, metabolism, and first‐pass disposition of dietary polyphenols and other phytochemicals in vivo. Future LC–MS/MS studies will therefore be needed to determine the systemic and joint‐tissue exposure of the prioritized compounds (Iwata 2024).

Second, MD simulations utilized a single 100‐ns trajectory per complex. This sampling window may miss rare dissociation events or the full conformational landscape; thus, the observed CD38‐coumestrol and MAPK8‐coumestrol interactions do not preclude late‐stage dissociation or metastability. Extended multi‐replica simulations are required to robustly sample these conformational spaces. Furthermore, the single‐ligand‐single‐receptor model inherently fails to capture the network‐level regulation driven by the complex TPC matrix in vivo (Cui et al. 2025).

Transcriptomic analyses are constrained by cohort composition. The training cohort exhibited class imbalance, and external validation sets were small. Despite restricting SMOTE to the training phase, AUC‐PR declined from 0.998 in the training cohort to 0.690 in Validation Set 2, reflecting attenuated cross‐cohort generalizability. Furthermore, reliance on synovial bulk‐transcriptomic data restricts these findings to the synovial compartment, precluding direct translation to peripheral blood or other clinically accessible tissues. Consequently, the six‐gene signature functions as a molecular prioritization tool, not a clinical diagnostic classifier.

Finally, the predicted compound‐target interactions lack direct experimental validation. Although single‐cell in silico perturbations mapped regulatory network shifts, physical coumestrol‐target engagement and ensuing cellular responses remain unmeasured. Future studies must integrate target‐specific perturbations with direct binding assays to definitively confirm these relationships (Lu et al. 2025; Tu et al. 2020). Ferroptosis‐related responses could be assessed using complementary cellular readouts, including lipid ROS, GPX4, and SLC7A11 (Zhao et al. 2023).

This study delineates a multilayer model wherein TPC‐derived polyphenols, notably coumestrol, modulate inflammatory, metabolic, and oxidative‐stress networks in RA. Structural analyses prioritize CD38 and MAPK8 as principal interaction targets, whereas PTGS2 retains robust transcriptomic and network‐level relevance despite dynamic instability during MD simulations. Single‐cell perturbations demonstrate that these targets drive distinct yet partially overlapping regulatory states. Decoupling physical binding from transcriptional and network‐level responses establishes a robust framework for prioritizing candidate mechanisms within the complex TPC matrix.

## 5 Conclusion

By integrating multicohort transcriptomics, machine learning, structural simulations, and single‐cell in silico perturbations, this study establishes a multidimensional network linking TPC‐derived polyphenols to inflammatory, metabolic, and oxidative‐stress pathways in RA. Coumestrol emerges as a bioactive constituent, with CD38 and MAPK8 receiving robust structural support as principal interaction targets. PTGS2 exhibits prominent transcriptomic and network‐level relevance despite dynamic instability during MD simulations, illustrating the divergence between transcriptional prominence and predicted ligand‐target binding persistence. Furthermore, single‐cell perturbations demonstrate that MAPK8 and PTGS2 mediate partially convergent regulatory states, whereas CD38 drives a distinct network response. These findings bridge structural dynamics with cellular network states, providing a prioritized molecular framework for experimental validation.

## Author Contributions

**Weiya Lan:** writing – original draft, methodology, visualization, conceptualization. **Qianwei Lu:** writing – original draft, methodology, visualization, software. **Xudong Rui:** data curation, investigation. **Ke Li:** data curation, investigation. **Chaoji Su:** validation, formal analysis. **Qiang Su:** validation, formal analysis. **Yanchang Huang:** supervision, visualization. **Run Shi:** visualization, supervision. **Wei Chen:** resources, project administration, writing – review and editing. **Fang Tang:** project administration, resources, writing – review and editing.

## Funding

This research was supported by Guizhou Provincial Science and Technology Department General Program (No. Qiankehe Jichu MS [2026] 694), Guizhou Provincial Clinical Research Center for Traditional Chinese Medicine in Rheumatic and Immune Diseases Project (Project Number: Qian Kehe Platform Talent [2020] 2202), Guizhou University of Traditional Chinese Medicine National and Provincial Science and Technology Innovation Talent Team Cultivation Project (Guizhou University of Traditional Chinese Medicine TD NO.[2022]004), The Second Affiliated Hospital of Guizhou University of Traditional Chinese Medicine “Sanhang” Talent Training Project for Scientific Research Talents (SHRC‐KY2024019), GUIZhongyi‐Jiao‐Gong‐He (2024) No. 95, Department of Education of Guizhou Province, The Guizhou Postgraduate Education Innovation Program (Grant No. 2024YJSKYJJ365).

## Conflicts of Interest

The authors declare no conflicts of interest.

## Supporting information


**Figure S1:** Protein–protein interaction (PPI) network of candidate targets.
**Figure S2:** Data normalization and sample distribution of the GEO training dataset (GSE89408).
**Figure S3:** Precision‐Recall (PR) curves for the optimized plsRglm model.
**Figure S4:** Evaluation of batch effect correction.
**Figure S5:** Network‐level regulatory displacement following virtual overexpression of MAPK8 and PTGS2.
**Figure S6:** Network‐level regulatory displacement following virtual knockout of CD38, MAPK8, and PTGS2.

**Table S1:** Significant regulatory responses identified by single‐cell in silico perturbation analyses.

**Data S1:** Raw SwissADME evaluation outputs for bioactive components.

## Data Availability

The processed expression matrices, formatted machine‐learning input datasets, R code for the 175‐model ensemble pipeline, model‐configuration file, and molecular‐dynamics files supporting this study are publicly available in the GitHub repository: https://github.com/QianWei‐Lu/TPC‐RA‐computational‐reproducibility. The original transcriptomic datasets are publicly available from the NCBI Gene Expression Omnibus under accession numbers GSE89408, GSE55235, GSE55457, GSE55584, GSE77298, GSE206848, and GSE200815.
