# Supplementary material for: Computational Prediction of the Therapeutic Mechanisms From the Turmeric‐Pueraria‐Coix Seed Botanical Formula in Rheumatoid Arthritis
Source: Food Sci Nutr. 2026 Oct 21;14(10):e72503. doi: 10.1002/fsn3.72503 (PMC13685524; doi:10.1002/fsn3.72503)
Supplement: Supplementary file 1 [file FSN3-14-e72503-s003.docx]

**Supplementary Fig. 1 Protein-protein interaction (PPI) network of candidate targets.**

Nodes represent individual target proteins, and the connecting lines indicate known or predicted functional interactions between them.

**Supplementary Fig. 2 Data normalization and sample distribution of the GEO training dataset (GSE89408).**

(A) Boxplot illustrating the distribution of normalized gene expression levels [Log2(CPM + 1)] across all individual samples in the Normal (blue) and RA (red) groups. (B) Principal component analysis (PCA) plot demonstrating the global transcriptional variance and spatial clustering of samples between the two groups.

**Supplementary Fig. 3 Precision-Recall (PR) curves for the optimized plsRglm model.**

PR curves served as a complementary metric to ROC analysis due to class imbalance in the training cohort (152 RA vs. 28 HC). SMOTE was applied during model training to mitigate this imbalance but omitted from external validation cohorts. The model achieved AUC-PR values of 0.998, 0.930, and 0.690 in the training cohort, Validation Set 1, and Validation Set 2, respectively. The reduced AUC-PR in Validation Set 2 reflects attenuated predictive performance across independent cohorts.

**Supplementary Fig. 4 Evaluation of batch effect correction.**

Principal component analysis (PCA) plots visualizing sample distributions (A) before and (B) after batch effect adjustment. Distinct experimental groups are denoted by different colors and shapes (Normal: red circles; RA: blue triangles). The altered clustering patterns in the post-correction plot reflect the mitigation of technical variations.

**Supplementary Fig. 5. Network-level regulatory displacement following virtual overexpression of MAPK8 and PTGS2.**

Top 20 genes ranked by regulatory displacement fold change following virtual overexpression of MAPK8 (A) and PTGS2 (B) in the RA synovial single-cell regulatory network. Burgundy bars indicate genes meeting the multiple-testing threshold (FDR < 0.05), whereas teal bars indicate non-significant responses (FDR ≥ 0.05). Regulatory displacement fold change reflects changes in network position between the baseline and perturbed manifolds and should not be interpreted as conventional gene-expression fold change.

**Supplementary Fig. 6. Network-level regulatory displacement following virtual knockout of CD38, MAPK8, and PTGS2.**

Top 20 genes ranked by regulatory displacement fold change following virtual knockout of CD38 (A), MAPK8 (B), and PTGS2 (C) in the RA synovial single-cell regulatory network. Burgundy bars indicate genes meeting the multiple-testing threshold (FDR < 0.05), whereas teal bars indicate non-significant responses (FDR ≥ 0.05). For MAPK8 and PTGS2 knockout, the perturbed target itself was retained in the visualization to show the magnitude of the imposed perturbation but was excluded when summarizing downstream regulatory responses. Regulatory displacement fold change represents network-level manifold displacement rather than conventional differential-expression fold change.
